# The influence of consumer awareness in the digital era on the selection of smartphones: A study among Jordanian university students

**DOI:** 10.1016/j.heliyon.2024.e25635

**Published:** 2024-02-08

**Authors:** Sami Alsmadi, Khaled Qassem Hailat, Saleh Bazi, Hadeel B. al-Haddad

**Affiliations:** Department of Marketing, Faculty of Business, Yarmouk University, Jordan

**Keywords:** Brand preference, Price perception, Product features, Purchase intention, Social influence, Smartphones

## Abstract

This research investigated and critically evaluated Jordanian university students' smartphone buying intentions. It critically reviewed relevant literature and conducted a focus group (Study 1), which empirically yielded a streamlined list of smartphone purchase criteria. Data for the primary study (Study 2) was obtained from university students (*n* = 427). The primary research hypotheses and significant factors were examined using structural equation modeling (SEM). Social impact (influence), product price, brand image, product features, perceived utility (usefulness), ease of use, and product support service were all found to influence the intention to acquire a smartphone, but at different strengths. Social impact (influence) had the least effect, whereas ease of use, product support service, and product price had the most significant effect. The current findings implied that rationality was likely to characterize Jordanian customers' smartphone purchases. These findings may enhance smartphone marketers’ efforts to improve their products and promotional activities. Technology-based product purchases showed a sensible trend among Jordanian university students as this was bolstered by paying less attention to “social influences” on such buying behavior. Clearly, Jordanian university students were using logical thinking when making technology-based product purchases. Furthermore, consumer culture and demographics' effects on technological product purchases should be studied in the future research.

## 1 Introduction

Today, smartphones represent an important benchmark of modern telecommunication technology as they are rapidly developing and increasingly influencing consumers in their daily routine behavior. Smartphones are become an indispensable part of our everyday lives, whether they are in our hands or always close at hand in our pockets. These devices, which emerged nearly three decades ago, facilitated almost all types of borderless communications among people across the world. Hence, the smartphone industry plays an essential role in today's borderless world [1,2]. For example, smartphones with internet communication enable people to stay in touch with business, friends, family, emails, social media communications, etc. Furthermore, the modern smartphone has a high technical capability of using operating systems to run variety of software programs. In fact, the huge variety of smartphone devices with different specifications available in the market give consumers a wide range of choices to satisfy their communication needs [3,4]. The proliferation of smartphone technology assisted in business processes, reduced cost, and improved overall work efficiency [5,6]. Since 2008, both the size of the smartphone market and the number of models and companies making them have grown steadily. In 2022, the world shipped about 1.2 billion smartphones. By the end of the year 2022, 68 percent of the people in the world were using smartphones. But since many people have more than one smartphone device, the number of subscriptions to smartphones is higher than the number of smartphone users. As of 2022, there were about 6.5 billion smartphone users. By 2028, that number is expected to reach almost 8 billion [7]. Jordan’s smartphone market is among the top most penetrated in the world as 78.1 % of the Jordanian users have internet connectivity via their smartphones [8]. Smartphones have further become a life partner for many customers, who use them numerous times each day to undertake a broad variety of social, cognitive, emotional, and business tasks. In the modern business world and society, smartphones have also become an integral part of everyday life.

Generally, it is noticed that consumers use smartphones with multimedia and multiple functions, such as calling, entertaining, playing music, multimedia messaging, internet browsing, and social media communication. On the other hand, Jordanian network service providers sell variety of services with varying specifications in their target markets [9]. However, there is a limited knowledge on the factors that motivate Jordanian university students to purchase smartphones continuously. Existing literature is mostly quantitative and empirically-based on information technology and systems theories. To the best of our knowledge, no previous studies have researched into the Jordanian context. Previous literature tackled China [10], South Korea [11], Taiwan [12] and many more.

The increasing prevalence of smartphones in everyday life has profound implications for Jordanian society [13]. Understanding the factors influencing the purchase intentions of smartphones among Jordanian university students is crucial given the societal impact of these devices on communication, education, and social interactions [14]. The smartphone market is dynamic and highly competitive. Investigating the purchasing behavior of a specific demographic, such as Jordanian university students, contributes valuable insights for smartphone manufacturers and marketers [15]. This information can aid in the development of targeted strategies to meet the unique preferences and needs of this consumer group. While there may be existing research on consumer purchase intention, our study focuses on a specific demographic in a unique cultural and educational context. This contributes to the academic literature by providing a nuanced understanding of factors influencing purchase decisions among Jordanian university students.

The current research adopts a mixed method research, where study-1 investigated the factors that drove Jordanian university students to purchase smartphones, and study-2 validated the research model in study-1. This triggered the main research question in the current study, which asks: *to what extent do cultural factors, including preferences for certain features or brands, influence consumer purchase intention of a smartphone among Jordanian university students?* To address this research question, the current study investigated key factors, which were frequently cited in the literature as determinants of a consumer purchase intention of a smartphone, drawing on the case of university students in Jordan.

## 2 Literature review

### 2.1 Smartphone technology adoption theories

As smartphones have become ubiquitous in modern society, understanding the factors influencing their adoption is essential for researchers, practitioners, and policymakers. The review focuses on three prominent theories: the Technology Acceptance Model (TAM) [16], the Unified Theory of Acceptance and Use of Technology (UTAUT) [17], and the Diffusion of Innovations (DoI) theory [18]. By examining these theories and their applications. TAM is one of the most influential theories in the field of information systems research. Developed by Davis [16], TAM posits that perceived usefulness (PU) and perceived ease of use (PEOU) are critical determinants of users' intention to adopt and utilize technology. Numerous studies have applied TAM to investigate smartphone adoption, highlighting the importance of PU and PEOU in shaping users' attitudes and behaviors towards smartphones. UTAUT is a comprehensive model that integrates and extends several existing theories. UTAUT proposes that performance expectancy, effort expectancy, social influence, and facilitating conditions collectively influence users' behavioral intentions and technology adoption. Several studies have utilized UTAUT to examine the adoption of smartphones, demonstrating its effectiveness in influencing users' intentions and actual usage. DoI theory, proposed by Rogers in 1962, explains how and why innovations spread through a population over time. This theory identifies five characteristics of innovations that influence their adoption: relative advantage, compatibility, complexity, trialability, and observability. DoI has been widely employed to study smartphone adoption, revealing the significance of these characteristics in driving adoption decisions. These theories have been tested widely in influencing smartphones purchase intention [[19], [20], [21], [22]].

### 2.2 Smartphone purchase intention

Several studies examined key factors affecting consumer-buying behavior pertaining to a smartphone purchase intention from different perspectives and in different places of the world. Below is a brief account of several leading studies on this topic in different countries. In Malaysia, several studies were conducted on this topic. For example, Mokhlis and Yaakop [23], who studied Malaysian university students, found that the three most important criteria influencing smartphone choice behavior were innovative product features, price, and word of mouth. Similar studies recommended word-of-mouth (social influence) for consideration in promoting smartphones among university students [24,25]. In the same vein [26], concluded that peer influence, self-innovativeness, self-efficacy, attitude, budget, age, and gender were significant factors that influenced demand for smartphone purchase by Malaysian consumers. Kim, Chun [27] found that signal affiliation and timely technology adoption were key determinants of a smartphone buying intention by American consumers.

In Indonesia, several similar studies were conducted. One study found a significant and positive relationship between brand image and smartphone purchase intention [28]. A study by Ref. [29] unveiled that country of origin and perceived quality were likely to play a significantly positive role in a smartphone purchase intention of Indonesian consumers. A study of Indonesian university students by Soriton and Tumiwa [30] revealed that technology factors, usability features, perceived quality, perceived ease of use, and brand loyalty were key determinant factors in a smartphone purchase decision. Rafdinal and Senalasari [31] concluded that ethnocentrism and perceived value were significant in influencing Indonesian consumer behavior of buying Chinese smartphone products. Chun, Matsumoto [32] investigated Indonesian and Japanese consumers, found that perceived risk, consumer innovativeness, and price were all vital factors affecting a smartphone purchase intention in both countries. Similarly, one study concluded that social needs, social influence, and convenience were significantly affecting a smartphone purchase intention among Indonesian university students [33].

In India, many similar studies were conducted on this topic. For example, an exploratory study by Malviya, Saluja [34] investigated the influence of four independent factors, namely price, brand preference, social influence, and product features, and revealed that only price was not significant, whereas the other three variables were significant in determining a smartphone purchase intentions among Indian consumers. Iqbal, Khan [35] found that electronic-word of mouth (e-wom) credibility, quantity, and format were influential in the purchase intention of a smartphone. The average lifetime for a smartphone is 2.67 years [36].

In North America, several studies addressed this topic. For example, one study by Negahban and Chung [37] investigated a smartphone choice behavior among university students and revealed that perceived ease of use, perceived usefulness, perceived enjoyment, and symbolic value are the determinants. Bringula, Moraga [38] unveiled that trust, purchase experience, and capability are important consideration in a purchase of a smartphone.

In many other countries, several similar studies were conducted in the same vein from different perspectives. In Bangladesh, Ali, Karim [39] found out that five variables were found significant in this buying behavior, namely Brand trust, reference group, service quality, reasonable price, and perceived usefulness. In Oman, Belwal, Hoque [40] found that students’ preferences for smartphones changed over time, not only for brands but also for the way they used these products and their related services. In Filipino, A study by Castillo, Flores [41] found that Filipino consumers were focusing on product brand and brand equity in their purchase intention of a smartphone. In Saudi Arabia, A study by Aldhaban, Daim [42] concluded that performance expectancy construct, effort expectancy, brand influence, perceived enjoyment and design determined a smartphone purchase intention. An Iranian study found that High-quality consciousness, brand consciousness, novelty-fashion consciousness, price consciousness, impulsiveness and carelessness, choice confusion, habitual and brand orientation, store location, recommendations and criticism by others, and power of parents were key determinants in the purchase intention of smartphones [43]. In Turkey, a study of university students conducted by Avcilar and Alkevli [44] found that perceived ease of use, perceived usefulness, perceived enjoyment, mobile shopping flow experience, mobile shopping attitude, mobile shopping satisfaction, perceived utilitarian value, and hedonic value were primary factors in a smartphone purchase intention. An Ethiopian study by Sata [45] showed that price, social influence, durability, brand name, product feature and after-sales service were influential factors in this a smartphone buying behavior. In Finland, Karjaluoto, Karvonen [46] found that innovative services, multimedia design, brand name, product features, outside influence, product reliability, and price were potential determinants in the purchase of a smartphone. Table 1 shows a summary of the above studies.

**Table 1:** A summary of factors that influenced consumer purchase intention of a smartphone.

Author(s)	Country	Factors Influencing a smartphone purchase intention.
Yusuf and Abd Rashid [47]	Malaysia	Price, social influence, relative advantage, and brand image.
Mokhlis and Yaakop [23]	Malaysia	Innovative product features, price, and personal recommendation (word of mouth).
Savitri, Hurriyati [28]	Indonesia	Brand image.
Prahiawan, Fahlevi [29]	Indonesia	Country of origin and perceived quality.
Soriton and Tumiwa [30]	Indonesia	Technology factors, usability features, perceived quality, perceived ease of use, and brand loyalty.
Rafdinal and Senalasari [31]	Indonesia	Ethnocentrism and perceived value.
Chun et al. (2022).	Indonesia/Japan	Perceived risk, consumer innovativeness, and price.
Poan, Hardi [33]	Indonesia	Social needs, social influence, and convenience.
Malviya, Saluja [34]	India	Brand preference, social influence, and product features.
Iqbal, Khan [35]	India	E-WoM credibility, quality, and format.
Negahban and Chung [37]	USA	Hedonism derived from using smartphones and individual’s dependence on smartphones.
Bringula, Moraga [38]	USA	Trust, purchase experience, and capability.
Ali, Karim [39]	Bangladesh	Brand trust, reference group, service quality, reasonable price, and perceived usefulness.
Castillo, Flores [41]	Filipino	Product brand and brand equity.
Mao, Lai [48]	China	Brand image, brand communication, brand identity, brand personality, flow experience.
Nasimi, Pali [43]	Iran	Quality consciousness, brand consciousness, novelty-fashion consciousness, price consciousness, impulsiveness and carelessness, choice confusion, habitual and brand orientation, store location, recommendations and criticism by others, and power of parents
Avcilar and Alkevli [44]	Turkey	Perceived ease of use, perceived usefulness, perceived enjoyment, mobile shopping flow experience, mobile shopping attitude, mobile shopping satisfaction, perceived utilitarian value, and hedonic value
Sata [45]	Ethiopia	Price, social influence, durability, brand name, product feature and after-sales service
Karjaluoto, Karvonen [46]	Finland	Innovative services, multimedia design, brand name, product features, outside influence, product reliability, and price.

Clearly, the above studies were conducted in different places and addressed the topic under study from various perspectives. These studies, which were frequently cited in the literature, revealed a variation in the number and type of factors to consider in a smartphone purchase intention. A careful examination of these studies (Table 1) revealed that these variations were possibly due to cultural and socio-economical differences in markets, in addition to variations in the history of smartphones in different markets. For example, some studies indicated only few criteria with less details for consideration in a smartphone purchase intention in certain countries (for example, Savitri, Hurriyati [28]). Whereas other studies examined several factors as key determinants in a smartphone purchase intention in other places of the world (for example, Ali, Karim [39]).

However, due to these variations in the number and type of these factors, a focus group of consumers and marketing experts was conducted to narrow down and specify the key factors to examine and analyze in this study as potential determinants in a smartphone purchase intention. The results of this focus group were further enhanced by a pilot study (next section).

## 3 Research methodology

### 3.1 Study 1: a qualitative research approach

Study 1 was qualitative in nature and designed to build new framework. It was determined that a qualitative methodology based on focus group was the most appropriate procedure [49]. Specifically, focus groups were used because they are well-suited for gaining an extensive understanding of a group's attitudes, experiences, emotions, perceptions, beliefs, norms, and values [50]. Importantly, focus groups reveal not only the group's dominant perspective on a given issue, but also the various perspectives that exist within the group [51]. The focus groups were conducted using a phenomenological methodology [52]. This method is ideal for describing an everyday phenomenon or activity from a particular group's perspective [53]. In addition, focus groups are optimal for comprehending student participants as a result of the insights obtained through discussion [54].

The propose of this focus group was to discuss all factors, which were examined in the previous studies as key factors affecting a smartphone purchase intention in the Jordanian context, in order to reach a potential list of factors for using in the current study, and possibly exclude irrelevant factors. The group moderator screened a comprehensive list of factors in the first session, which triggered a discussion and strong debate in the group. The focus group consisted of a convenient sample of customers, mainly university students (10 students), and consumer behavior specialist (5 members). Brainstorming discussion and creative thinking continued throughout all sessions. Three sessions were conducted before a final concluding session was reached. Each session lasted about 60 min, with a different moderator for each session to avoid potential bias.

The focus group data was analyzed using discursive psychology approach, as outlined by Edwards and Potter [55]. This approach was chosen due to the psychological nature of brand addiction, as individuals experience it on a psychological level. Discursive psychology, as described by Potter [56], investigates the processes by which individuals construct, understand, and disclose psychological phenomena within their daily experiences. The analysis in this study adhered to the recommended procedure outlined by Jonathan and Wetherell [57] and Carla [58]. Firstly, the recordings were transcribed using Jefferson's [59] method of transcription, as described by Potter [56]. Next, relevant sections of the transcripts were selected based on thematic categories that were linked to the research questions, following the guidelines provided by Jonathan and Wetherell [57]. The transcripts were then coded to identify sections that required further analysis, drawing on the approach outlined by Silverman [60]. Selected excerpts of the coded transcripts were analyzed in relation to the research questions and focal research issues, as recommended by Carla [58]. Finally, the main themes were identified through inferential interpretations of the data, employing conversational sequencing techniques, management of turn-taking, and analysis of talk overlap and repair, as described by Hutchby and Wooffitt [61].

The data analysis of the focus group revealed the following seven factors as key determinants (purchase criteria) in a smartphone purchase intention in the Jordanian context:

(1) Social influence
(2) Product Price
(3) Brand Image
(4) Product features
(5) Perceived usefulness
(6) Ease of use
(7) Product support service

**Fig. 1:**
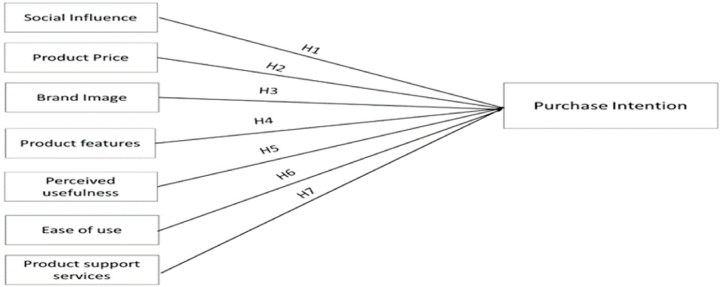
Conceptual model.

To enhance the findings of this focus group, a pilot study of academics and experts was further conducted to check the face validity of these seven factors and their measuring statements through a specifically developed questionnaire to collect the required data for the current study. The findings of the pilot study included some amendments on the measuring statements. Fig. 1 illustrates the ***conceptual model*** which reflected the above seven factors that were assumed to influence a smartphone buying intention in the Jordanian context. The current study hypotheses were set in accordance with this model, as illustrated in the next paragraph.

#### 3.1.1 Research hypotheses

In view of the above conceptual model, and the review of literature and discussions of the key seven factors influencing a smartphone purchase intention in the Jordanian context, the following hypothesis were set for further testing in the data analysis stage:

H1 There is a statistically significant and positive impact of social influences on the customer's purchase intention of a smartphone

H2 There is a statistically significant and positive impact of product price on the customer's purchase intention of a smart phone.

H3 There is a statistically significant and positive impact of brand image on the customer's purchase intention of a smart phone.

H4 There is a statistically significant and positive impact of product features on the customer's purchase intention of a smartphone.

H5 There is a statistically significant and positive impact of Perceived usefulness on the customer's purchase intention of a smartphone.

H6 There is a statistically significant and positive impact of Ease of use on the customer's purchase intention of a smartphone.

H7 There is a statistically significant and positive impact of Product support service on the customer's purchase intention of a smartphone.

#### 3.1.2 Construct/variables operationalization and measures

**Social Influence**: One type of social influence is WOM. The social influence that exerts psychological pressure on the buying behavior of a customer. It often causes a change in our buying behavior either intentionally or unintentionally. It usually results from the direct and indirect interaction with other people (friends, peers, acquaintances, relatives, family members, media, and social media interaction). Four-item were adapted from Yusuf and Abd Rashid [47].

**Product Price**: Price is often a key concern for consumers when making a purchase decision [62]. Consumers usually build internal reference prices over time. This occurs, as consumers are exposure to product prices in the market. When they notice a price tag, they often compare it to their reference price. Four measurement items were adapted from Yusuf and Abd Rashid [47].

**Brand Image**: a brand is defined as “a name, term, sign, symbol, or design, or combination of them, intended to identify the goods or services of one seller or group of sellers and to differentiate them from those of competitors” Keller [63]. Brand preference occurs when a consumer consistently prefer a brand to other brands. This brand becomes a consumer’s first choice behavior. Three-items were adapted from Savitri, Hurriyati [28] to measure brand image.

**Product Features**: product attributes that often lead to consumer benefits, which deliver value to consumers and differentiate a product in the market. Five-item were adapted from Mokhlis and Yaakop [23].

**Perceived Usefulness**: it describes perceived benefits that a customer can derive from a product attributes. Five-item were adapted from Davis [16] to measure perceived usefulness construct.

**Ease of use:** it describes the perceived ease of using a product by a customer. Three-item were adapted from Davis [16] to measure perceived ease of use.

**Product Support Service:** it is the technical product support provided by a marketer. For example, it includes after-sale services, maintenance, parts, and warranty. Five-item were adapted to measure product support service from Sata [45].

**Purchase Intention**: In the classical model of consumer decision making, purchase intention is established to proceed to the buying process, which goes through problem recognition, information search, evaluation, purchase, and post-purchase behavior [64]. In the context of this study, purchase intention precedes a purchase decision for a smartphone. Five items were adapted from Dodds, Monroe [65] to measure purchase intention.

### 3.2 Study 2: validating the research model

#### 3.2.1 Study population and sampling procedures

The current study investigated a target population of university students (all levels) studying at Yarmouk university-the second largest university in the north of Jordan, with a student body totaling 40,000 students from 45 different nationalities, distributed over 16 Colleges (https://www.yu.edu.jo/). An initial convenience sample of 500 students was drawn from this population, using Drop-and-Collect method Malhotra, Wu [66]. According to this method, the field workers conducted short personal interviews with students, through which they hand delivered the questionnaires to them to fill out, with an arrangement to collect the completed questionnaires later on the same day. The useable sample was (*n* = 427) bringing the response rate to 85.4 %. The target population in this study was considered relatively homogeneous in terms of age group and education level for obvious reasons. Therefore, age and education variables were removed from the study as they were assumed fixed. Gender and income (family income) were the two demographic variables considered in this study. Table 2 shows the distribution of the study sample according to gender and income.

**Table 2:** Sample demographics.

Variable	Variable level	Frequency	Percentage (%)
Gender	Male	258	60.4
	Female	169	39.6
Household income	Below JD500	203	47.5
	JD500–JD1000	161	37.7
	JD000	63	14.8

Note: *n* = 427; JD: Jordanian Dinar (Jordan currency).

#### 3.2.2 Research instrument

A structured, self-administered, questionnaire was developed to measure the level of influence of the seven factors that were assumed to affect a smartphone purchase intention among university students at Yarmouk University, as shown earlier. This questionnaire was approved by the ethical committee of Yarmouk university-the IRB committee (Institutional Reviewing Board), including the informed consent of survey participants. The statements that were used to measure these factors were adapted from previous studies as follows:

(1) Social Influence:
  SI1: Remarkable people use smartphones.
  SI2: Important people use smartphones.
  SI3: Most of the people around me use smartphones.
  SI4: Most of smartphone users are role models in society.
(2) Product Price:
  PR1: Price is an indicator of the quality of a smartphone.
  PR2: I attract attention of others when I buy an expensive smartphone.
  PR3: Buying an expensive smartphone makes me feel good about myself.
  PR4: Buying an expensive smartphone makes me socially attractive.
(3) Brand Image:
  BI1: I always care about the mental image of a smartphone brand.
  BI2: My smartphone’s brand enjoys a wide reputation.
  BI3: My smartphone’s brand is characterized by originality.
(4) Product Features:
  PF1: When buying a smartphone, I pay attention to the power of its software.
  PF2: When buying a smartphone, I pay attention to its lightweight.
  PF3: When buying a smartphone, I pay attention to its shape attractiveness.
  PF4: When buying a smartphone, I pay attention to its level of security protection.
  PF5: When buying a smartphone, I pay attention to its color.
(5) Perceived Usefulness:
  PU1: Using a smartphone makes online shopping easier.
  PU2: Using a smartphone helps me keep in touch with my emails.
  PU3: Using a smartphone helps me do my work easier.
  PU4: Using a smartphone helps me keep in touch with others.
  PU5: Using a smartphone helps me do my work efficiently.
(6) Ease of Use:
  EU1: In general, using a smartphone is easy.
  EU2: Using a smartphone does not require much mental effort.
  EU3: Dealing with various applications through a smartphone is easy.
(7) Product Support Service:
  PS1: The availability of technical support service of smartphones is satisfactory.
  PS2: The technical support service for smartphones is provided quickly.
  PS3: The level of warranty provided for smartphones is satisfactory.
  PS4:The availability of spare parts for smartphones is satisfactory.
  PS5: The after-sales service of smartphones is satisfactory.
(8) Purchase intention
  PI1:The likelihood of purchasing this product is.
  PI2:If I were going to buy this product, I would consider buying this smartphone at the price shown.
  PI3: At the price shown, I would consider buying the product.
  PI4: The probability that I would consider buying the product is.
  PI5: My willingness to buy the product is.

The questionnaire included seven parts (1–7) to measure **Purchase intention**. An additional part was added to assess the demographic variables of respondents (gender and income). The questionnaire was translated to Arabic language for using in data collection, as the population’s main language was Arabic. The translation was double-checked by two translators independently to ensure accuracy.

A five-point Likert scale was used for measuring the statements representing the (7) main factors. The scale uses five levels of agreement (from strongly disagree up to strongly agree). The second and the fourth items of purchase intention were scaled by the five levels of likeability (from very high likeable to very low likeable). The higher the attitude mean score the more favorable the attitude, and vice versa.

#### 3.2.3 Analysis procedures

In view of the critical review of the literature, the researchers believed that there could be possible relationships among and within the factors affecting the purchase intention of a smartphone. To evaluate how well the proposed model represents the observed data, a Structural Equation Model (SEM) technique was used to analyze the collected data [67,68]. The measurement model, which attempted to validate the utilized measurements and examine their psychometric qualities, was calculated first, followed by the structural model for testing the hypotheses. A confirmatory factor analysis (CFA) was undertaken to examine the criteria for reliability, content validity, convergent validity, and discriminant validity [69]. As part of the CFA analysis, a common method bias test (CMB) was performed to evaluate if the data had measurement bias. After setting validity and CMB criteria, the hypotheses were evaluated using a structural equation model. Maximum Likelihood Method (MLM) estimated the structural model. IBM SPSS and AMOS (V.29) were used for estimation requirements.

### 3.3 Data analysis

#### 3.3.1 Common method bias (CBM) and measurement model (CFA)

To examine the multicollinearity, a common method bias (CMB; [70]) analysis was used to check for bias in the data. Standardized estimates were calculated for both the CLF (Common Latent Factor) model and the non-CLF model to see whether there was a significant difference in factor loadings (0.05). The findings showed that the discrepancy ranged from (0.012) to (0.036) with no (CMB) issues with the data [71].

CFA results confirmed model constructs, supporting their continuing usage in the structural model assessment. Cronbach's alpha and composite reliability ratings (Table 3, Table 4) were both more than (0.70), indicating that the scale was reliable and internally consistent [72]. Table 3 revealed that the factor loadings were larger than (0.60), which fulfilled the content validity criteria [73]. However, three elements (SI3, PR1, PI1, and PI5) were removed due to weak factor loadings. The convergent validity criterion was met since the AVE values were more than (0.50), as shown in the table [74].

**Table 3:** Results of CFA.

Construct	Item	Item-Factor loadings*	CA	CR	AVE
Social Influence	SI1	0.845	0.752	0.882	0.714
	SI2	0.864			
	SI4	0.826			
Product Price	PR2	0.882	0.850	0.928	0.812
	PR3	0.925			
	PR4	0.895			
Brand Image	BI1	0.923	0.814	0.906	0.763
	BI2	0.832			
	BI3	0.864			
Product Features	PF1	0.832	0.773	0.917	0.690
	PF2	0.815			
	PF3	0.835			
	PF4	0.864			
	PF5	0.806			
Perceived usefulness	PU1	0.754	0.838	0.897	0.637
	PU2	0.805			
	PU3	0.824			
	PU4	0.795			
	PU5	0.812			
Ease of use	EU1	0.879	0.736	0.891	0.733
	EU2	0.936			
	EU3	0.885			
Product support service	PS1	0.832	0.825	0.939	0.756
	PS2	0.856			
	PS3	0.875			
	PS4	0.882			
	PS5	0.902			
Purchase Intention	PI2	0.895	0.872	0.946	0.854
	PI3	0.932			
	PI4	0.945			

Note: CFA: Confirmatory Factor Analysis; CA represents “Cronbach’s Alpha”; CR: Composite Reliability, AVE represents “Average Variance Extracted”; CFA Fit indices: ϰ2/df = 4.276; CFI = 0.948 (Good fit>0.9); GFI = 0.93,; RMSEA = 0.048 (Good fit <0.06); TLI: 0.915; *: denotes p < 0.05.

**Table 4:** Discriminant validity.

Construct	Brand Image	Ease of use	Perceived usefulness	Product Price	Product support services	Product Features	Purchase intention	Social Influence
Brand Image	0.847							
Ease of use	0.178 (0.216)	0.825						
Perceived usefulness	0.092 (0.265)	0.344 (0.229)	0.628					
Product Price	0.433 (0.566)	0.270 (0.303)	0.121 (0.239)	0.683				
Product support services	0.284 (0.389)	0.221 (0.229)	0.289 (0.510)	0.221 (0.325)	0.728			
Product Features	0.477 (0.728)	0.158 (0.583)	0.225 (0.441)	0.282 (0.464)	0.241 (0.411)	0.609		
Purchase intention	−0.049 (0.068)	0.101 (0.120)	0.126 (0.108)	−0.042 (0.071)	0.054 (0.082)	0.095 (0.110)	0.695	
Social Influence	0.298 (0.431)	0.237 (0.311)	0.348 (0.519)	0.309 (0.596)	0.236 (0.397)	0.303 (0.543)	0.127 (0.108)	0.676

Note: The numbers in the orthogonal show the AVE, and the numbers in the brackets show the HTMT ratio.

Table 4 revealed that each independent variable created a diagonal (AVE) bigger than the inter-correlation with the other constructs. Every single requirement for validity matched the standards that were established by Fornell and Larcker [74] and Bagozzi, Li [75]. Regarding the evaluation of the Heterotrait-Monotrait (HTMT) ratio, no construct correlations were found greater than the threshold value of (0.85) as defined by Henseler, Ringle [76]. This indicated that the discriminant analysis was valid. The footnote in the Table explained a summary of the model's fit capacity to explain the data. The fit indices indicated a satisfactory fit in the measurement model based on established criteria [67,69].

Based on previous research, along with scale development, focus group, and pilot study efforts, the conceptual model for this study suggested seven latent variables, each of which was related to observable variables as previously defined (see research instrument section). The analysis already showed that the measurement model was appropriate for the collected data and ready for hypothesis testing. All further investigations relied on the standard estimations already established. The measurement model was applied to each scale at the same time. Factor loadings were meticulously reviewed to assess convergent validity according to Bollen’s [77] recommendations. Both the factor loadings and squared multiple correlations values were greater than (0.7), indicating satisfactory convergent validity.

The sample size of the current study (*n* = 427) is comparable to the sample size recommended for statistical analysis using SEM [68]. The fit statistics in the current study indicated an acceptable fit of the measurement concerning the sample data based on recommended values [78]. As determined by the CFA model fit analysis, the parameters were within acceptable ranges (Table 3).

#### 3.3.2 Hypotheses testing

**Fig. 2:**
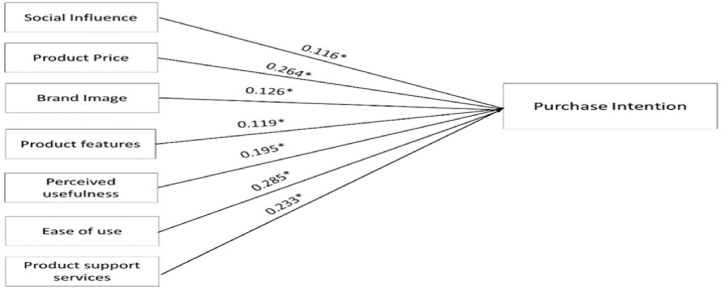
The structural model results. Note: *: 0.05 significant level.

The structural equation model was performed through AMOS (V29). Fig. 2 illustrated the hypothesized model, which presented the dependent and independent variables, including the hypothesized relationship with the hypotheses directions between dependent and independent variables in the specified model. Table 5 showed the structural model assessment as outlined in Fig. 2. Overall, the exogenous variables together significantly explained (42.5 %) of the variation in the purchase intention variable (dependent variable), and that all the hypotheses (H1–H7) were significantly supported. The results in the Table revealed that The ‘ease of use’ factor demonstrated the highest impact on the purchase intention as its path coefficient was (0.285), followed by ‘Product Price (0.264)’ and ‘Product support services (0.2.33)’ respectively. While the ‘social influence’ factor showed the least impact on the purchase intention with a path coefficient value of (0.116).

**Table 5:** SEM results.

Hypotheses	Exogenous Variable	Endogenous Variable	Path Coefficients*	Hypotheses supported	R^2^
H1	Social Influence	Purchase intention	0.116	YES	0.425
H2	Product Price		0.264	YES	
H3	Brand Image		0.126	YES	
H4	Product features		0.119	YES	
H5	Perceived usefulness		0.195	YES	
H6	Perceived ease of use		0.285	YES	
H7	Product support services		0.233	YES	

Note: SEM Fit indices: ϰ2/df = 4.252 GFI = 0.921, CFI = 0.935, (Good fit >0.9); RMSEA = 0.051 (Good fit <0.06); TLI: 0.910; *: denotes p < 0.05.

## 4 Discussion

The research paper explores the factors influencing the purchase intention of smartphones among Jordanian university students. It highlights the significance of smartphones in modern lifestyle and their growing influence on consumers' daily routine behavior. The study's main research question is to identify the factors likely to influence the purchase intention of smartphones among this specific demographic in Jordan.

This study examined the role of multiple factors in influencing the intention to buy a smartphone device among Jordanian university students. The findings revealed that the ‘ease of use’ was playing the strongest role in establishing the buying intention of a smartphone. In the dynamics of consumer behavior, this pattern looks reasonably rational when a consumer deals with technologically sophisticated products like smartphones. The current findings showed that the second most influential factor was the ‘product support services’ factor. The price of a smartphone represented the third factor affecting the intention of buying smartphones. Looking at the whole picture, these three factors were interacting together to influence consumer behavior rationally to think of buying a smartphone. In a way, this indicted that Jordanian university students were likely to show a rational buying behavior when buying technologically sophisticated products. This conclusion was further enhanced by the fact that Jordanian university students were paying less attention to the ‘social influence’ factor in developing such a buying intention. This provides a clear evidence to believe that Jordanian university students behave rationally when they develop buying intentions to buy sophisticated products.

Smartphone purchase decisions are evidently influenced by a wide range of factors, including but not limited to the consumers' perceived symbolic and cultural meanings of smartphone brands [10] and the brands' ability to communicate these meanings [79]. These results contribute to our growing knowledge of smartphone consumer behavior. The factors are consistent with prior findings in other countries. Social influence was found to affect purchase intention [33,34,47], price [23,32,45], brand image [23,45], product features [23,34,46], perceived ease of use, usefulness [30,43], and product support service [39,45]. However, they varied in the extent of their influence on purchase intention.

### 4.1 Theoretical contributions

The present research contributes to the existing literature on consumer purchase intentions of smartphones in several ways. Firstly, it explores the factors that influence Jordanian university students' intentions to purchase smartphones, filling a significant gap in the literature. While previous studies investigated smartphone purchase intentions in various countries. This study is the first to focus on Jordan, providing insights into the specific contextual factors that shape consumer behavior in this unique market.

Secondly, the present research draws on three prominent theories, namely the TAM, UTAUT, and the DoI to explain the factors affecting smartphone purchase intentions. By integrating and adapting these theories to the Jordanian context, the study enhances our understanding of how technology adoption and innovation diffusion theories apply in different cultural and socio-economic settings.

Thirdly, the study enriches the literature on smartphone purchase intentions by identifying the specific determinants that are most relevant to Jordanian university students. By conducting focus group discussions and pilot studies, the research narrows down and specifies the key factors that influence purchase intentions in Jordan, which may differ from those in other countries due to cultural, economic, and historical variations. This localized understanding allows for more targeted marketing strategies and interventions in the Jordanian smartphone market.

Finally, the integration of TAM, UTAUT, and DoI theories in this study provides a comprehensive framework for analyzing smartphone adoption and purchase intentions. This framework can be applied in other contexts and markets to gain a deeper understanding of consumer behavior in the smartphone industry.

### 4.2 Practical implications

The findings of this research have several practical implications for smartphone manufacturers, marketers, and policymakers in the Jordanian context. First and foremost, understanding the factors that drive smartphone purchase intentions among Jordanian university students can help smartphone manufacturers tailor their products to meet the specific needs and preferences of this consumer category. For example, emphasizing features that are most valued by Jordanian university students, such as innovative product features, brand trust, and social influence, could enhance the appeal of smartphones in this market.

Secondly, marketers can leverage the insights from this study to design more effective marketing campaigns that resonate with the Jordanian university students’ mindset. Highlighting the factors that were identified as influential in smartphone purchase intentions, such as product quality, perceived value, and convenience, can lead to higher consumer engagement and better brand perception.

Furthermore, policy makers and industry stakeholders can use the research findings to develop targeted initiatives to promote smartphone adoption and usage in Jordan. For instance, understanding the significance of ethnocentrism and perceived value in influencing consumer behavior can inform policies to encourage the purchase of locally manufactured smartphones, supporting the domestic smartphone industry.

## 5 Limitations and future research

Despite its contributions, this study has some limitations that provide avenues for future research. Firstly, the research focused specifically on Jordanian university students, which may limit the generalizability of the findings to the broader population. Future studies could expand the sample to include a more diverse demographic to obtain a more comprehensive understanding of smartphone purchase intentions across different age groups and socio-economic backgrounds.

Secondly, the qualitative nature of Study 1, although valuable in exploring in-depth perspectives, might be limited in its ability to quantify the relative importance of different factors. Future research could employ a larger-scale quantitative survey to provide more statistically robust insights into the strength of the relationships between factors and smartphone behavior rather than purchase intention, like, customer engagement, brand equity, customer equity, and financial performance.

Lastly, the study was conducted at a specific point in time, and the smartphone market is continually evolving. Future research should consider conducting longitudinal studies to track changes in consumer preferences and behavior over time, allowing for a deeper understanding of the dynamic nature of the smartphone industry. Despite these limitations, this research represents an important step in understanding the factors.

## 6 Conclusion

This study specifically addresses the smartphone purchase intentions of Jordanian university students. Utilizing the TAM, UTAUT, and DoI theories, we tailored our investigation to this demographic group, offering a thorough understanding of the factors influencing their decisions in the context of Jordan's unique cultural and economic landscape.

Our findings are particularly relevant to the academic discourse and practical applications within Jordan. The adaptation of renowned technology adoption theories to the Jordanian context enriches the localized literature and provides actionable insights for stakeholders within the country. The practical implications of this study are tailored to meet the specific needs and preferences of Jordanian university students. Smartphone manufacturers, marketers, and policymakers can leverage these insights to develop strategies that resonate with this demographic group, enhancing smartphone adoption and usage.

While our findings offer a foundation for understanding smartphone purchase intentions, they are particularly contextualized for Jordanian university students. As technology and consumer preferences evolve, stakeholders within Jordan can utilize this research as a foundational resource for informed decision-making to address consumer needs within this specific context.

## Funding

The authors declare that no funds, grants, or other grants were received for the preparation of this manuscript.

## Data availability statement

Data associated with this research paper has not been deposited into a publicly available repository. Data will be available upon request.

## CRediT authorship contribution statement

**Sami Alsmadi:** Writing – review & editing, Writing – original draft, Conceptualization. **Khaled Qassem Hailat:** Methodology, Data curation. **Saleh Bazi:** Validation, Methodology, Formal analysis. **Hadeel Hadad:** Writing – review & editing, Writing – original draft.

## Declaration of competing interest

The authors declare that they have no known competing financial interests or personal relationships that could have appeared to influence the work reported in this paper.
